# Long-range near-surface wake signatures of offshore wind farm clusters revealed by satellite observations

**DOI:** 10.1038/s44172-026-00684-7

**Published:** 2026-05-15

**Authors:** Rui Li, Jincheng Zhang, Xiaowei Zhao

**Affiliations:** Intelligent Control & Smart Energy (ICSE) Research Group, School of Engineering, University of Warwick, Coventry, UK

**Keywords:** Wind energy, Energy modelling

## Abstract

Wind energy has expanded rapidly in recent years, leading to increasingly dense clusters of offshore wind farms. As a result, wind farm wake effects, manifested as reduced wind speeds downstream of operating turbines, have become an important consideration for wind resource assessment and large-scale planning. Here we investigate near-surface wake signatures using wind speeds retrieved from 7122 Sentinel-1A/B synthetic aperture radar (SAR) images acquired between 2020 and 2022, covering more than 60 offshore wind farms across Europe and Asia. A consistent processing workflow and an automated wake-detection algorithm are applied to identify wake-affected regions and quantify wake-related wind speed changes at 10 m height. The results show that near-surface wake signatures can persist over distances exceeding 100 km under favorable conditions and that wake-affected regions exhibit an average wind speed reduction of 0.990 m/s (12.4%) at 10 m height. Several cases of wake interactions extending across national boundaries are also observed in densely developed offshore regions. These findings provide a large-scale, observation-based characterization of near-surface wind farm wake signatures.

## Abstract (web summary)

Rui Li and colleagues report widespread near-surface wake effects across offshore wind farms using thousands of satellite images. The results show that wakes can extend beyond 100 km, highlighting implications for large-scale wind energy planning.

## Introduction

Wind energy is under sustained and accelerated development in recent years, driven by the soaring energy prices across the globe and the imminent net-zero target to tackle climate change^1–3^. Compared with their onshore counterparts, offshore wind farms offer numerous advantages, including higher wind velocities with reduced turbulence, enhanced social acceptance, greater space availability, and heightened energy density owing to the feasibility of deploying larger turbines^4^. When selecting an appropriate site for constructing a new wind farm, various design factors should be considered, including wind resource potential, initial construction cost, operation and maintenance expenses, and proximity to transmission lines and roads^5,6^. However, finding sea areas that meet all these requirements can be challenging. Consequently, offshore sites with favorable wind conditions, soil quality, and water depth, such as the North Sea and the Yellow Sea, are witnessing the proliferation of closely spaced wind farms^7,8^. For example, dozens of large-scale wind farms owned by different countries are already operating in the North Sea, with more farms planned for the future^9^. As these sea areas become increasingly crowded, it is crucial to consider and quantify the wake effects, which refer to the reduction in wind speeds in the downstream region caused by upstream wind farms. Understanding and addressing these wake effects is essential to navigate legal issues and conflicts that will inevitably arise from uncoordinated wind farm development in the foreseeable future, thereby enhancing the sustainability in wind energy planning and utilization^10,11^.

Wind farm wakes, as a complex aerodynamic phenomenon, are influenced by many factors such as upstream wind speeds, atmospheric stability, and meteorological conditions. Currently, two mainstream technologies are used to investigate the potential impacts of wake effects: numerical simulations^12–16^ and in-situ measurements^17–21^. For example, high-resolution microscale and urban climate models, such as Parallelized Large-Eddy Simulation Model (PALM)^22–26^ and Microscale Transport and Stream Model (MITRAS)^27–29^, have demonstrated the capability to resolve wind farm wake dynamics and turbine-induced flow modifications at fine spatial scales. At the same time, a series of in-situ measurement campaigns^17,19,30,31^, including mast- and LiDAR-based observations, have provided direct observational evidence of wake effects in both onshore and offshore environments. However, numerical simulations cannot encompass the full range of real-world operating conditions, as they are constrained by prescribed boundary conditions and parameterizations. Conversely, in-situ measurements are inherently limited in spatial coverage, restricting their ability to capture wake behavior across multiple wind farms and broad geographic regions. Although previous observational and modeling studies have documented wake extensions exceeding 50-70 km under certain atmospheric conditions^2,17,32^, a systematic, multi-site, observation-based assessment of the statistical prevalence and spatial extent of near-surface wake signatures across large offshore wind farm clusters remains limited. This creates a gap between detailed local-scale studies and large-scale, observation-based characterization of near-surface wind-field modifications induced by wind farms, which satellite observations are uniquely positioned to address.

This study aims to address the aforementioned questions by utilizing Synthetic Aperture Radar (SAR) images to comprehensively investigate the impact of wind farm wakes. SAR images provide a reliable and universal means to analyze near-surface wind speeds in offshore environments, as the 10 m wind speed can be retrieved from these images using the C-band geophysical model function^33^. The reliability and consistency of SAR-derived wind speeds for offshore wind farms under both free-stream and wake conditions have been previously validated^34^. SAR images, captured by satellite-borne sensors, offer the advantage of global coverage, enabling the characterization of near-surface wake signatures worldwide with high fidelity. Although SAR images have been used to investigate wake effects in previous studies^14,32,35–40^, the scale of SAR data and the range of research areas have been limited, leaving the full potential of remote sensing technology untapped for wind energy applications.

**Fig. 1:**
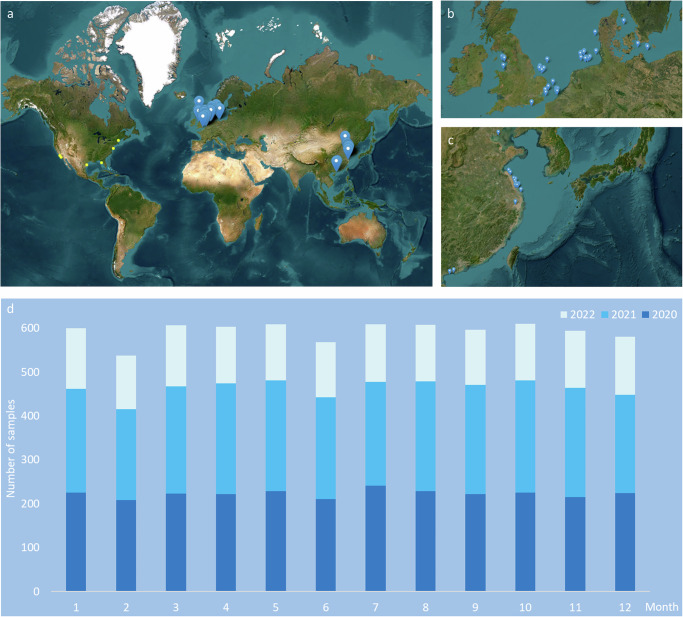
Spatial and temporal distribution of Sentinel-1A/B SAR acquisitions. **a** Geographic distribution of wind farms included in this study; yellow markers near the United States indicate buoy locations used for validation of SAR-derived wind speeds. **b** Distribution of European wind farms. **c** Distribution of Asian wind farms. **d** Monthly sample counts from 2020 to 2022. Map Credit: The satellite imagery layer is the intellectual property of ESRI and is used herein under license (Copyright © ESRI and its licensors. All rights reserved). The underlying geographic basemap data is sourced from OpenStreetMap and is open access, freely available under the Open Data Commons Open Database License (ODbL) (© OpenStreetMap contributors).

In this work, we collect a comprehensive dataset consisting of 7122 Sentinel 1A/B SAR images, totaling more than 11.5 TB of data, obtained from the Copernicus Open Access Hub over a three-year period (2020–2022). These images cover over 60 offshore wind farms across Europe and Asia. The spatial and temporal distributions of the wind farms analyzed in this study are illustrated in Fig. 1. Only SAR images captured after the wind farms were fully commissioned are utilized to ensure a consistent evaluation. Furthermore, the data collection spans evenly all months of the year, as shown in Fig. 1d, allowing for the examination of seasonal variations in wake behavior. Based on the collected data, a processing pipeline is developed to retrieve the wind speed while the accuracy is verified by the buoy data. Finally, an image-processing method is proposed to obtain the upstream freestream wind speeds and the downstream wake speeds.

The wind farm wake effects have profound economic implications globally and are acknowledged as a crucial concern by both academia and industry. However, neither the government nor the wind energy industry has established a settlement or legislation to compensate downstream wind farms for the revenue loss caused by upstream wind farm wakes yet^5,10^. This lack of consensus can be attributed, in part, to the absence of a measurable and quantifiable index to assess the real economic impact of wake effects. The evidence provided by this work from SAR-derived wake-induced near-surface wind speed deficit assessment may accelerate the policy-making processes by providing technical support to address such issues. By analyzing thousands of Sentinel-1 SAR scenes across multiple offshore wind farm clusters, this study bridges the gap between isolated case studies and multi-regional statistical confirmation of near-surface wake signatures.

## Methods

### Wind speed retrieved from the SAR images

**Fig. 2:**
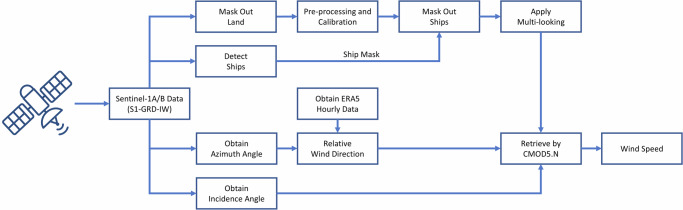
Workflow of SAR-based wind speed retrieval. Sentinel-1A/B GRD (IW mode) data are first processed to mask out land and ships, followed by calibration and multi-looking. Ship detection is performed to generate a ship mask for removing contamination. The azimuth and incidence angles are extracted from SAR data. ERA5 hourly data are used to derive the relative wind direction. These parameters are then used as inputs to the CMOD5.N geophysical model function to retrieve near-surface wind speed.

In this study, we utilize the CMOD5.N algorithm^33^ to retrieve wind speeds from the SAR images. The entire retrieval process is summarized in Fig. 2. Initially, we collect Sentinel-1A/B SAR images from the Copernicus Data Space Ecosystem (https://browser.dataspace.copernicus.eu/), with the product type being GRD and the sensor mode set to Interferometric Wide (IW). Subsequently, we apply pre-processing and calibration using the Sentinel Application Platform (SNAP) toolbox (http://step.esa.int/main), including thermal noise removal, orbit correction, radiometric calibration and speckle noise filtering. Furthermore, we mask out land areas and remove bright objects like turbines and ships, as they tend to create strong radar reflections in the SAR image. The resulting images are then subjected to multi-looking, resulting in a final resolution of 500 meters. For wind speed retrieval using CMOD5.N, three external parameters are required: azimuth angle, incidence angle and wind direction. The first two parameters could be directly obtained from the SAR images themselves. Regarding wind direction, ECMWF Reanalysis version 5 (ERA5) hourly data (https://www.ecmwf.int/en/forecasts/dataset/ecmwf-reanalysis-v5)^41^ are used as a reference. By combining all these procedures, the wind speed could be eventually retrieved from the SAR images. The details of each pre-processing step are provided in Table 1.

**Table 1:** The details of the pre-processing procedure for SAR images

Procedure	Details
Thermal noise removal	The backscatter disturbances caused by thermal noise are corrected, generating more seamless SAR images.
Orbit correction	The orbital information is updated in the SAR data using a more accurate Orbit State Vector (OSV) file, which is selected as Sentinel Precise in this work.
Radiometric calibration	The reflectivity of SAR is converted into physical units of normalized backscatter by normalizing the reflectivity using a reference plane.
Speckle noise filtering	The Refined Lee^57^ method is adopted to remove speckles and smooth the grainy salt and pepper effect.
Bright object detection	The adaptive thresholding function embedded within the SNAP with a Two-Parameter Constant False Alarm Rate (CFAR) Detector is adopted. The target size, target/guard/background window sizes and Probability of False Alarm (PFA) (10^−*x*^) are set as (30, 300), (10, 350, 600) and 12.5.
Multi looking	50 by 50 multi-looking operation is conducted in this procedure.

### Verification of retrieved wind speed using buoy data

To assess the accuracy of the wind speed retrieved from the SAR images, we conduct a verification process using buoy data provided by the National Data Buoy Center (NDBC) (https://www.ndbc.noaa.gov). We collect data from six different NDBC buoy stations as shown in Fig. 1a and corresponding SAR images from the same locations spanning the period from 2020 to 2022. The buoy station IDs and their respective coordinates are as follows: 42020 (26.968N, 96.693W), 46025 (33.755N, 119.045W), 44009 (38.460N, 74.692W), 44007 (43.525N, 70.140W), 42036 (28.501N, 84.508W) and 46086 (32.499N, 118.052W). Next, we process the SAR images using our developed C-band model pipeline to retrieve the wind speed. We then extract the wind speed values at the corresponding buoy locations based on their coordinates and compare them to the buoy data. In total, we obtain 945 matched pairs of buoy data and retrieved wind speeds (with 117, 304, 46, 84, 77 and 317 pairs for each station, respectively). Since most of the NDBC anemometers are installed at a height of 2.5–4 m above the sea surface, we convert the measured speeds to the 10-m wind speed using the logarithmic laws^42^:

1 $${u}_{({z}_{r})}=\frac{{{{\rm{In}}}}\frac{{z}_{r}}{{z}_{0}}}{{{{\rm{In}}}}\frac{z}{{z}_{0}}}{u}_{(z)},$$

where ${u}_{({z}_{r})}$ and *u*_(*z*)_ represent the wind speeds at the height *z*_*r*_ and *z*, respectively. *z*_0_ is the surface roughness, which was set to 0.0002 m for the sea surface^43,44^.

**Fig. 3:**
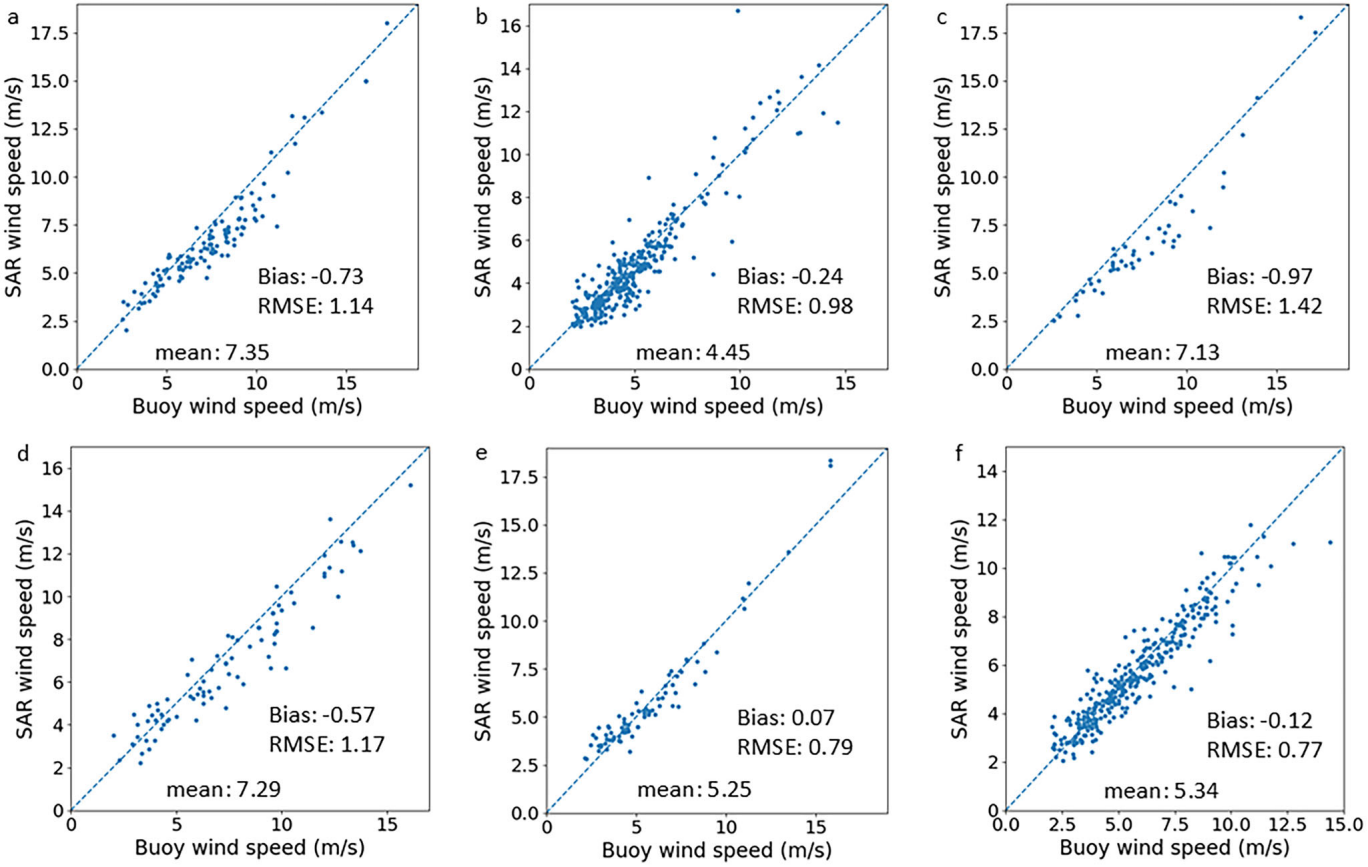
Comparison of SAR-retrieved wind speed and NDBC buoy wind speed. Scatter plots show Sentinel-1A/B SAR wind speeds retrieved using CMOD5.N versus buoy wind speeds from the National Data Buoy Center (NDBC). The dashed line denotes the 1:1 relationship. Bias, root mean square error (RMSE), and mean buoy wind speed are shown in each panel. **a**–**f** correspond to six buoy sites.

The scatter plots depicting the comparison results for the six cases can be seen in Fig. 3, while detailed statistical metrics are reported in Table 2. The comparison results reveal that the root mean square errors (RMSE) for the six cases are 1.14 m/s, 0.98 m/s, 1.42 m/s, 1.17 m/s, 0.79 m/s, and 0.77 m/s, respectively, indicating that the retrieved wind speeds from the SAR images exhibit agreement with the buoy data. While SAR-derived wind speeds may not perfectly reproduce point-scale measurements, their accuracy and spatial consistency are sufficient for analyzing relative wind speed variations and wake-induced deficits over large offshore areas, which is the primary focus of this study.

**Table 2:** Multi-year verification of Sentinel-1 10-m wind speeds against in-situ buoy observations

Buoy	*N*	RMSE	Bias	MAE	*R*	SI	RMSE 95% CI	Bias 95% CI
		(m s^−1^)	(m s^−1^)	(m s^−1^)			(m s^−1^)	(m s^−1^)
Buoy1	117	1.14	−0.73	0.93	0.95	0.15	[1.00, 1.29]	[−0.89, −0.56]
Buoy2	304	0.98	−0.24	0.68	0.92	0.20	[0.81, 1.16]	[−0.34, −0.13]
Buoy3	46	1.42	−0.97	1.10	0.95	0.18	[1.11, 1.70]	[−1.27, −0.68]
Buoy4	84	1.17	−0.57	0.93	0.95	0.15	[0.97, 1.35]	[−0.79, −0.36]
Buoy5	77	0.79	+0.07	0.59	0.96	0.14	[0.62, 0.93]	[−0.11, +0.25]
Buoy6	317	0.77	−0.12	0.56	0.94	0.13	[0.68, 0.85]	[−0.20, −0.03]

Reported metrics include number of collocations (*N*), root-mean-square error (RMSE), mean bias (SAR–buoy), mean absolute error (MAE), Pearson correlation coefficient (*R*), scatter index (SI), and bootstrapped 95% confidence intervals (CI) for RMSE and bias.

### Upstream and downstream wind speed extraction

**Fig. 4:**
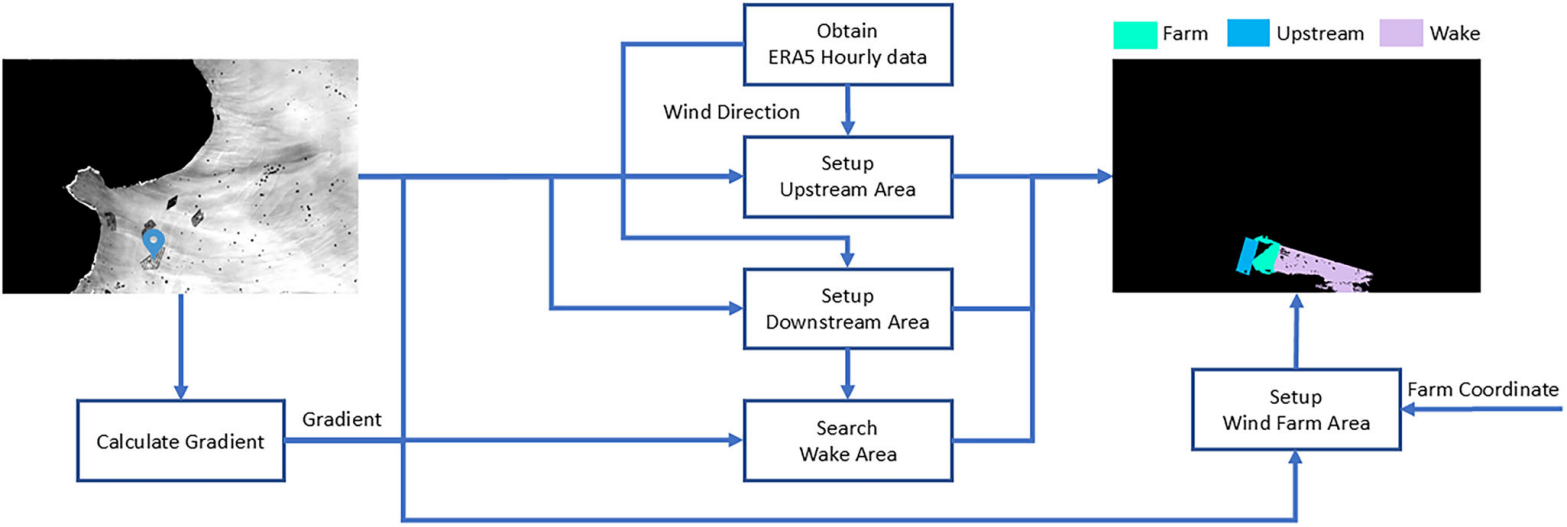
Workflow of the proposed *R*^2^*G*^3^ for upstream and wake-affected areas detection. Sentinel-1 SAR data are first used to calculate the spatial gradient of wind speed. ERA5 hourly data are employed to determine wind direction, which is then used to define upstream and downstream regions relative to the wind farm. Based on these regions, a search is conducted to identify wake-affected areas using gradient information. The wind farm area is delineated using known farm coordinates. The method integrates gradient-based analysis and geometric constraints to detect wake-affected regions. Colored regions represent the wind farm (green), upstream (blue), and wake-affected (purple) areas. Credit of the source SAR image: European Space Agency (ESA).

In this study, we develop an image processing method called Restricted-Region Gradient-Guided Growing (*R*^2^*G*^3^) to distinguish between the upstream free speed and the downstream wake speed from the retrieved wind speed data. During the pre-processing procedure, bright objects such as turbines and ships are masked out, which results in small or zero wind speed values in those areas after the multi-looking procedure, leading to abnormally high gradients. Similarly, areas affected by the wake effect typically have lower wind speeds compared to their surroundings, resulting in higher gradients at the boundary between wake and non-wake areas. Based on the above intuition, we calculate the gradient of the retrieved wind speed image to guide the identification of the Region of Interest (ROI) corresponding to the upstream and downstream areas, as shown in Fig. 4. Obviously, the real ROIs are the upstream and downstream areas, excluding the wind farm itself. To achieve this, we use the location of the wind farm as the initial point and identify adjacent pixels with abnormal gradients as the farm area. Then, the local wind direction is extracted from the ERA5 hourly data^41^, which helps determine the orientations of the upstream and downstream areas. We assume that the upstream and downstream areas are rectangular in shape, with heights of 20 pixels (approximately 10 km) and 100 pixels (approximately 50 km), respectively. The widths of the rectangles are manually set according to the scale of the corresponding wind farm. For the upstream area, we filter out pixels with abnormally high gradients or abnormally small wind speeds, and the remaining pixels are considered the final upstream area. For the downstream area, we start with a few pixels located downstream of the wind farm (without abnormal gradients) as initial seeds. Based on these seeds, we iteratively expand the downstream area by including pixels whose wind speed values are close to the average speed of the seeds. The average speed is updated with the inclusion of new pixels. Finally, we determine the wake area after checking all pixels in the predefined downstream search area. By using the *R*^2^*G*^3^ method, we can separate the upstream and downstream areas, allowing us to analyze and quantify the impact of near-surface wind farm wakes. The detailed procedure for detecting upstream and wake-affected areas is summarized in Algorithm 1 with default settings reported in Table 3, while the algorithm overview and sensitivity test of parameters are provided in Supplementary Notes 1 and 2.

**Table 3:** Default parameters used in the *R*^2^*G*^3^ wake extraction algorithm

Symbol	Description	Default value	Notes
**C**	Wind farm center coordinate	Farm centroid	Derived from turbine layout
*R*	Wind farm radius	Manually measured	Max distance from **C** to turbines
*D*	Wind direction	ERA5 10 m wind	Nearest time and location to SAR overpass
*L*_u_	Upstream length	10 km	Along-wind direction
*L*_d_	Downstream length	50 km	Along-wind direction
*W*	Upstream/downstream width	Set based on *R*	Cross-wind extent
*T*_1_	Lower wake speed ratio	0.6	*S*_*i*_≥*T*_1_ × *S*_ave_
*T*_2_	Upper wake speed ratio	1.1	*S*_*i*_≤*T*_2_ × *S*_ave_
*T*_u_	Minimum upstream speed	0.5 × *S*_ave_	Calculated based on the average waked wind speed
*G*_T_	Gradient threshold	2 × average gradient	Derived from pixels with gradient range (0.1, 50)
*N*	Initial downstream seed size	25 pixels	Center point + 3 offset layers {1, 3, 5} in 8 directions
${S}_{\min }$	Minimum valid wind speed	1 m/s	Avoids low-wind SAR uncertainty
${S}_{\max }$	Maximum valid wind speed	25 m/s	Avoids high-wind SAR uncertainty
${N}_{u,min}$	Minimum upstream area valid pixels	5 × *R*	Applied to *P*_u_
${N}_{d,min}$	Minimum wake area valid pixels	20 × *R*	Applied to *P*_d_

All parameters are applied uniformly to all SAR scenes unless otherwise stated.

#### Algorithm 1

*R*^2^*G*^3^ for upstream and wake-affected areas detection

**Require:** Wind Farm Coordinate **C**, Wind Direction *D*, Wind Farm Radius *R*, Upstream Length *L*_u_, Downstream Length *L*_d_, Wake Speed Threshold *T*_1_, *T*_2_, Upstream Speed Threshold *T*_u_

1: Calculate the gradient *G* of the wind speed field retrieved from the SAR image and set the threshold *G*_T_

2: Search pixels affected by wind turbines around **C** and exclude them from the subsequent analysis

3: Setup upstream area *A*_u_ based on *D*, *R* and *L*_u_ and initialize upstream point list *P*_u_ ← {}

4: Setup downstream area *A*_d_ based on *D*, *R* and *L*_d_

5: Initialize the average wake wind speed *S*_ave_ based on a point set *P*_d_ = {*P*_1_, *P*_2_, . . . , *P*_*N*_} within the *A*_d_

6: **for** *i* in *A*_d_ **do**

7: **if** *T*_1_ × *S*_ave_≤*S*_*i*_≤*T*_2_ × *S*_ave_ and *G*_*i*_≤*G*_T_ **then**

8: *P*_d_ ← {*P*_1_, . . . , *P*_*N*_, *P*_*i*_}

9: *S*_ave_ ← Ave(*P*_d_)

10: **end if**

11: **end for**

12: **for** *j* in *A*_u_ **do**

13: **if** *G*_*j*_≤*G*_T_ and *S*_*j*_≥*T*_u_ × *S*_ave_ **then**

14: *P*_u_ ← *P*_u_ ∪ {*P*_*j*_}

15: **end if**

16: **end for**

17: The final upstream area *P*_u_ and wake-affected area *P*_d_ are obtained.

## Results

All analyses are based on SAR-retrieved 10 m neutral wind speeds. The results, therefore, describe near-surface wake signatures and should not be directly interpreted as hub-height wind conditions. Based on the analysis of SAR images obtained from over 60 wind farms across Europe and Asia during the years 2020, 2021, and 2022, two findings regarding wind farm wake effects are presented in this study. Firstly, the study demonstrates that near-surface wake effects can extend more than 100 km. The extended wake range has important implications for wind farm planning and development. Secondly, the study reveals that on average, wind farm wake effects lead to a 12.4% reduction in 10 m wind speed in downstream wake-affected sea areas. While this reduction is quantified at the near-surface level, it may have implications for the power generation potential of downstream wind farms. The physical plausibility of the observed long-range and transboundary wake propagation is further supported by mesoscale simulations, as described in Supplementary Note 3.

### Long-range near-surface wake signatures confirmed by SAR

Early research utilizing SAR data has provided evidence that wind farm wakes can persist for more than 20 km downstream^35^. Mesoscale studies using the Weather Research and Forecasting (WRF) model have also shown velocity deficits in downstream areas at distances of at least 15 km from upstream wind farms^45^. However, with the increase in turbine size and farm scale, there is mounting evidence that estimates of 15 km or 20 km for wake propagation are no longer valid. In-situ measurements conducted over wind farms in the German Bight, such as Amrumbank West and Godewind, confirm that far wake effects can extend at least 45 km and can reach up to 70 km downwind^17^. Qualitative studies based on SAR images have also revealed that wakes can extend over 50 km^46,47^. In the case of superimposed wakes behind multiple wind farms, the wake length can reach distances of up to 70 km, as observed in SAR images^32^. While previous SAR-, LiDAR-, and model-based studies have reported long wind farm wakes under specific conditions, these investigations were largely limited to individual cases or regional domains. Comprehensive observational evidence documenting the spatial extent of near-surface wake signatures across multiple offshore wind farm regions has remained limited.

**Fig. 5:**
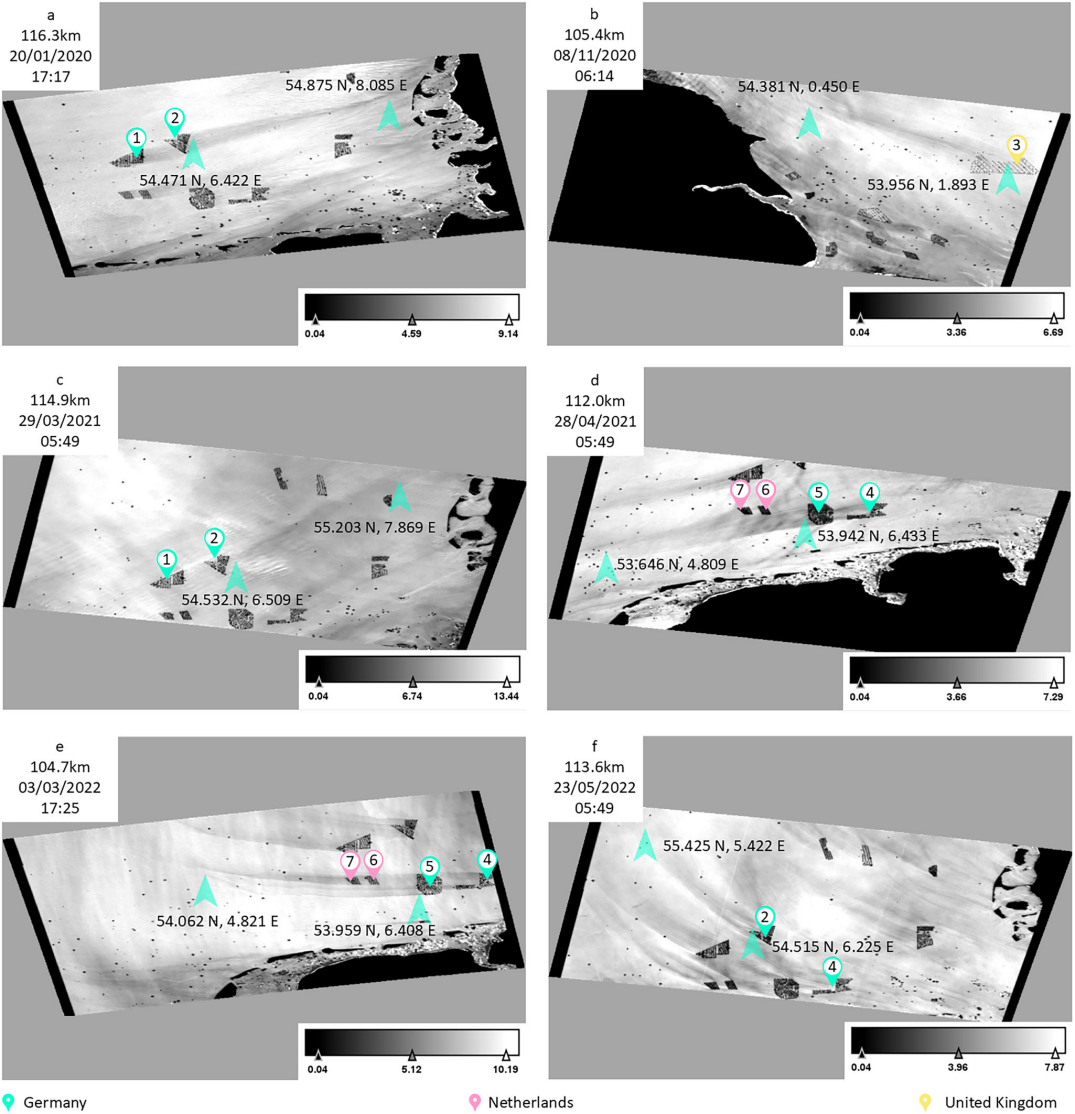
Examples of long-range near-surface wake signatures observed in SAR-derived 10 m wind speed fields. The grayscale color bars represent SAR-retrieved neutral wind speed at 10 m height (m/s), with darker tones indicating lower wind speeds and lighter tones indicating higher wind speeds. Blue arrows mark the manually identified start and end points used to estimate the wake propagation distance, which is calculated based on geographic coordinates. The acquisition date and time (UTC) are shown in each subpanel. The SAR scenes were captured: (**a**, **c**–**f**) in the German Bight; and (**b**) near the coast of Holderness and Yorkshire. The color of the wind farm location symbols represents the operating country (see legend at bottom). Detailed information on the wind farms is provided in Table 4. Credit of the source SAR images: European Space Agency (ESA).

Currently, the recovery of low momentum and high turbulence caused by upstream wind farms is deemed to require a distance of at least 50 km^5^. However, our study, based on SAR-retrieved wind speed, reveals observational evidence of long-range near-surface wakes extending up to about 100 km downstream under favorable atmospheric conditions, as shown in Fig. 5. These long-distance near-surface wakes were captured in the North Sea at various times over a three-year period. ERA5-derived bulk Richardson numbers were analyzed for these six wake cases. All events occurred under very stable boundary-layer conditions, indicating strongly suppressed turbulent mixing. This finding is consistent with the observed long wake persistence^17^, as reduced vertical momentum exchange delays wake recovery. The details of wind farms involved can be found in Table 4. Beyond the absolute wake extent, the SAR observations reveal that long-range wake persistence is strongly associated with wind farm clustering and cumulative installed capacity. In particular, near-surface wakes extending beyond 100 km predominantly occur where multiple large-scale wind farms are aligned with the prevailing wind direction, leading to superposition of individual wake signatures and delayed momentum recovery. This behavior is consistent with recent mesoscale modeling studies suggesting that wake recovery length scales increase nonlinearly with farm size and turbine density^48,49^, and the present results provide observational evidence consistent with these modeling results at regional scales. Meanwhile, the observation that a single ultra-large cluster, such as the Hornsea Project in Fig. 5b, can generate near-surface wake signatures exceeding 100 km further suggests that wake recovery assumptions commonly used in offshore planning may no longer be valid for next-generation wind farms. As turbine rotors increase in size and farms expand to gigawatt scales, wake-induced flow modification increasingly resembles a mesoscale phenomenon rather than a localized engineering effect.

**Table 4:** Information about wind farms contained in Fig. 5, including operating country, capacity (MW) and the number of turbines

Number	Wind Farm Name	Country	Capacity (MW)	Turbines
1	Deutsche Bucht Veja Mate BARD Offshore 1	Germany Germany Germany	252 402 402	31 67 80
2	Hohe See Albatros Global Tech I	Germany Germany Germany	497 112 400	71 16 80
3	Hornsea Project One Hornsea Project Two	United Kingdom United Kingdom	1218 1386	174 165
4	Nordsee One Gode Wind 1 Gode Wind 2	Germany Germany Germany	332.1 330 252	54 55 42
5	Trianel Windpark Borkum 1 Trianel Windpark Borkum 2 Borkum Riffgrund 1 Borkum Riffgrund 2 Merkur Alpha Ventus	Germany Germany Germany Germany Germany Germany	200 203 312 450 396 60	40 32 78 56 66 12
6 7	Gemini	Netherlands	600	150

In total, 18 wind farms belonging to three countries (Germany, the United Kingdom, and the Netherlands) are included, with 1269 turbines and 7.80 GW capacity.

At the same time, a considerable number of large-scale wind farms are currently being constructed or planned within the North Sea. For example, in the vicinity of wind farm cluster 1 in Fig. 5, several wind farms with a combined capacity of more than 14 GW are already under planning. At the same time, in the vicinity of cluster 2, multiple German wind farms with a combined capacity of nearly 3 GW are currently under preliminary assessment. As shown in Fig. 5a, c, the superimposed wakes caused by clusters 1 and 2 are already clearly visible and extend over a considerable distance. Upon completion of all the planned wind farms, with a capacity that is approximately nine times larger than the current capacity, the wake effects will cover a much wider geographical area and extend over a much greater distance. This substantial impact can have immediate economic repercussions and potentially hinder the development of future wind projects, reducing the sustainability of wind energy utilization. It becomes crucial to carefully consider and mitigate the potential challenges posed by these extended wake effects to ensure the sustainable growth and success of wind energy projects.

### Cross-boundary near-surface wake interactions revealed by SAR

In addition to the long propagation distance of wind farm wakes, the velocity deficits caused by wind farm wakes are also substantial. For onshore wind farms, an estimated power generation loss of 5% has been observed, based on an econometric model and numerical weather prediction simulations^5^. Simulation studies for offshore wind farms have reported velocity deficits of 7% extending 100 km downstream and 10% extending 80 km downstream^50^. Real-world observations have shown persistent wake effects with velocity deficits of approximately 21% at a distance of 55 km downstream, while the speed loss can reach as high as 41% approximately 24 km downstream, as measured by LiDAR^37^. Wake effects can even be observed at 200 m altitude, albeit with smaller deficits compared to the hub height measurements^51^.

Despite the extensive recognition and systematic investigation of wind farm wakes, the issue of justifying and quantifying their effects to resolve disputes between upstream and downstream wind farms remains unresolved^10,11^. The absence of solid evidence to demonstrate the influence of wake effects is an urgent issue that needs to be addressed. While simulation results can suggest that wake effects may decrease productivity under certain conditions, they cannot prove the actual impacts on downstream wind farms in real-world operating scenarios, not to mention when and how these impacts occur. In contrast, SAR images offer a valuable and generic solution, as they capture wind speeds under real-world operating conditions, providing strong observational evidence of the actual near-surface impacts of upstream wakes.

**Fig. 6:**
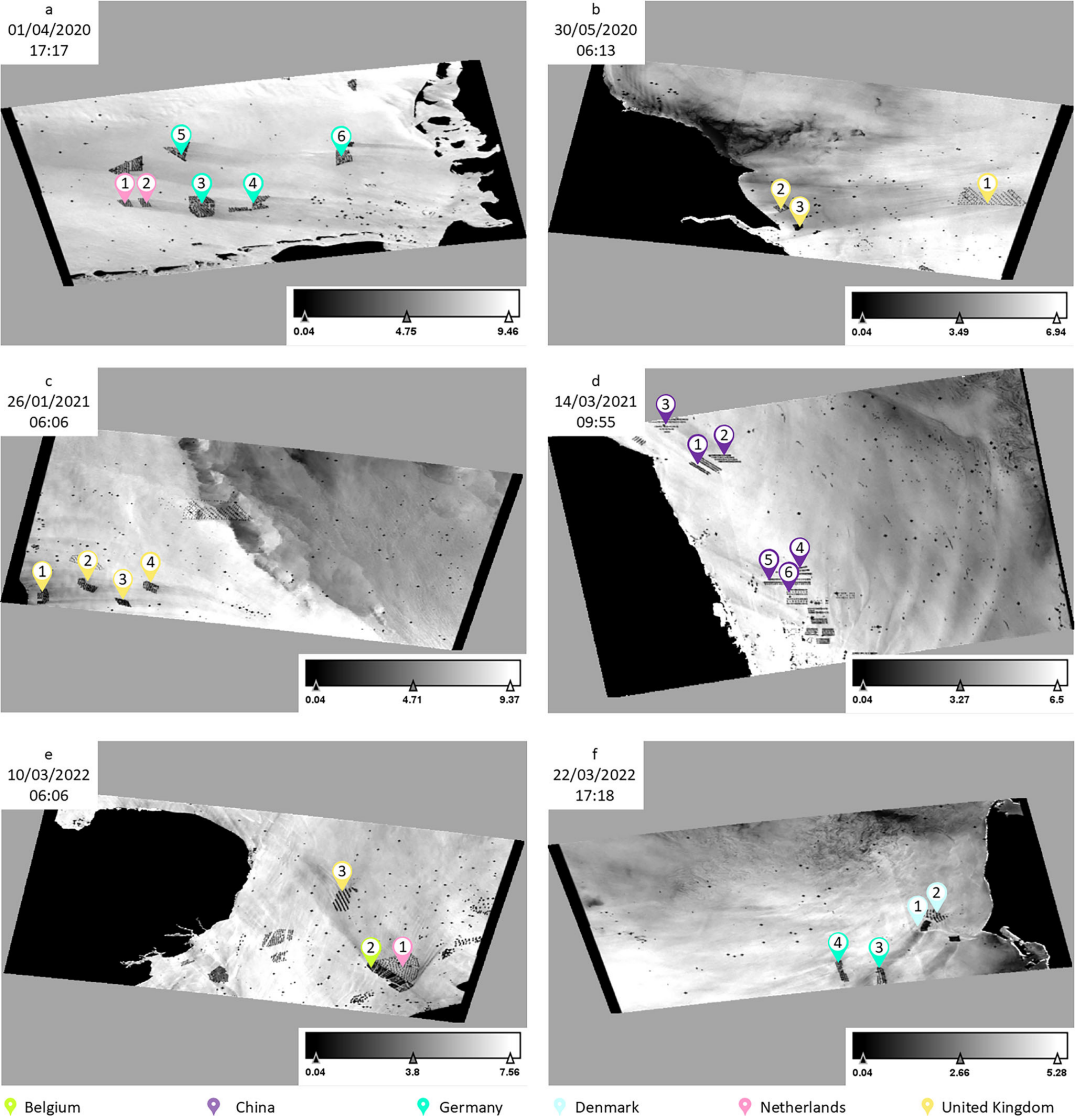
Examples of upstream wake impacts on downstream wind farms observed in SAR-derived 10 m wind speed fields. The grayscale color bars represent SAR-retrieved neutral wind speed at 10 m height (m/s), with darker tones indicating lower wind speeds and lighter tones indicating higher wind speeds. The acquisition date and time (UTC) are shown in the top-left corner of each subpanel. The color of the wind farm location symbols corresponds to the operating country (see legend at bottom). The SAR scenes were captured: **a** in the German Bight; **b** near the coast of Holderness and Yorkshire; **c** near the coast of Lincolnshire; **d** in the Yellow Sea near Jiangsu Province; **e** in the North Sea between the UK and the Netherlands; and **f** near the Danish Jutland coast. Detailed information on the wind farms involved is provided in Table 5. Credit of the source SAR images: European Space Agency (ESA).

Figure 6 visually represents the near-surface wake interactions between adjacent wind farms. Panels (b–d) demonstrate the wake interactions among wind farms within the same country, typically owned by different companies. In panel (b), the downstream wind farm clusters are entirely enveloped by the long-range and high-intensity near-surface wake effects generated by the upstream large-scale Hornsea Project. Medium-scale wind farms located in close proximity, as shown in panels (c, d), also experience substantial near-surface wake interactions with each other. Resolving disputes between domestic wind farms can be relatively straightforward with the involvement of local authorities. However, complexities arise when wake interactions occur between wind farms owned by different countries, as evident in panels (a), (e), (f). Taking panel (e) as an example, although three wind farm clusters are situated in their respective exclusive economic zones, the near-surface wake generated by clusters 1 (Netherlands) and 2 (Belgium) clearly affects downstream cluster 3 (United Kingdom). Table 5 provides detailed information about the wind farms involved in wake effects, including those generating wakes and those influenced by wakes. It is noteworthy that a wind farm cluster can be both a culprit and a victim depending on the wind direction. Resolving transnational wake interactions requires bilateral and multilateral negotiation and cooperation to mitigate potential conflicts. From a scientific perspective, these transnational wake interactions also highlight a limitation of site-specific wake models that neglect upstream developments beyond national boundaries. The SAR observations demonstrate that near-surface wake impacts can propagate across exclusive economic zones, implying that national-scale planning frameworks may systematically underestimate external wake exposure in densely developed sea basins such as the North Sea.

**Table 5:** Information about wind farms contained in Fig. 6, including operating country, capacity (MW) and the number of turbines

Panel	Number	Wind Farm Name	Country	Capacity (MW)	Turbines
a	1 2	Gemini	Netherlands	600	150
	3	Trianel Windpark Borkum 1	Germany	200	40
		Trianel Windpark Borkum 2	Germany	203	32
		Borkum Riffgrund 1	Germany	312	78
		Borkum Riffgrund 2	Germany	450	56
		Merkur	Germany	396	66
		Alpha Ventus	Germany	60	12
	4	Nordsee One	Germany	332.1	54
		Gode Wind 1	Germany	330	55
		Gode Wind 2	Germany	252	42
	5	Hohe See	Germany	497	71
		Albatros	Germany	112	16
		Global Tech I	Germany	400	80
	6	Meerwind Süd/Ost	Germany	288	80
		Nordsee Ost	Germany	295.2	48
		Kaskasi	Germany	342	38
b	1	Hornsea Project One	United Kingdom	1218	174
		Hornsea Project Two	United Kingdom	1386	165
	2	Westermost Rough	United Kingdom	210	35
	3	Humber Gateway	United Kingdom	219	73
c	1	Inner Dowsing	United Kingdom	97.2	27
		Lincs	United Kingdom	270	75
	2	Race Bank	United Kingdom	573.3	91
	3	Sheringham Shoal	United Kingdom	316.8	88
	4	Dudgeon	United Kingdom	402	67
d	1	Datang Jiangsu Binhai	China	300	95
	2	SPIC Binhai South H3	China	300	75
	3	SPIC Binhai North H2	China	400	100
	4	Huaneng Sheyang H1	China	301.5	67
	5	Longyuan Sheyang H2	China	301.5	67
	6	SPIC Jiangsu Dafeng H3	China	302.4	72
e	1	Borssele 1	Netherlands	376	47
		Borssele 2	Netherlands	376	47
		Borssele 3	Netherlands	351.5	37
		Borssele 4	Netherlands	380	40
		Borssele Site V	Netherlands	19	2
	2	Seamade (Mermaid)	Belgium	235	28
		Northwester 2	Belgium	219	23
		Nobelwind	Belgium	165	50
		Belwind	Belgium	165	55
		Seamade (SeaStar)	Belgium	252	30
		Northwind	Belgium	216	72
		Rentel	Belgium	309	42
		Norther	Belgium	369.6	44
		Thornton Bank - phase I	Belgium	30	6
		Thornton Bank - phase II	Belgium	184.5	30
		Thornton Bank - phase III	Belgium	110.7	18
	3	East Anglia ONE	United Kingdom	714	102
f	1	Horns Rev 2	Denmark	209.3	91
	2	Horns Rev 3	Denmark	406.7	49
	3	DanTysk	Germany	288	80
	4	Sandbank	Germany	288	72

In total, 52 wind farms related to wake effects (to influence or to be influenced) belonging to six countries (Belgium, China, Germany, Denmark, Netherlands and United Kingdom) are included, with 3004 turbines and 16.73 GW capacity.

### A first multi-regional census on wake effects

After identifying wake-affected regions within individual SAR scenes, this section focuses on conducting a large-scale statistical assessment to quantify the associated near-surface wind speed deficits.

By utilizing the retrieved wind speed data and identifying the upstream and wake-affected areas, this study accomplishes the first multi-regional census on wind farm wake effects. Initially, cases with very small detected areas (often due to farms located at the edge of the SAR image) and extreme wind speeds (above 25 m/s or below 1 m/s) are filtered out, resulting in 5920 samples for the subsequent quantitative assessment. Among all these samples, 3789 (64.0%) exhibit lower downstream wind speeds compared to the upstream wind speeds, indicating the presence of a near-surface wind speed deficit. As SAR is a real-world observation, there exist multiple potential factors that lead to the anomaly that the downstream wind speed is even higher than the upstream, such as the imaging quality, noise effect, side wind, retrieval quality and detection accuracy. And sometimes, this kind of phenomenon may indeed exist in the real world under certain atmospheric conditions^13,52–54^, which needs further in-depth investigation. As the real reasons are unclear, cases where the downstream wind speed exceeds the upstream wind speed are excluded from the statistical analysis to reduce ambiguity. This filtering step may influence both the distribution and magnitude of the reported wind speed deficits and should therefore be considered when interpreting the statistical results.

**Fig. 7:**
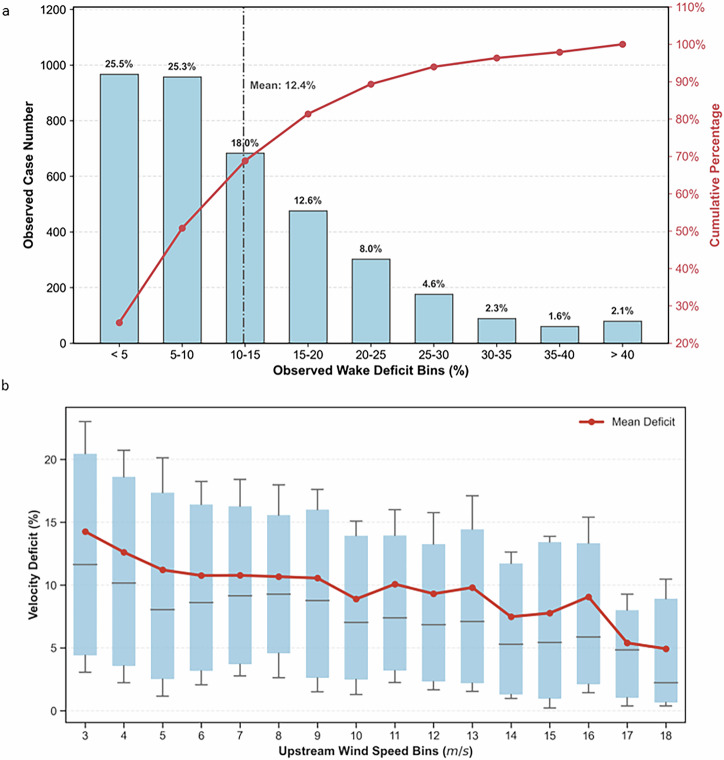
Distribution and characteristics of observed near-surface wind speed deficits. **a** Distribution of observed near-surface wind speed deficits grouped by percentage intervals. Bars represent sample counts and the red curve denotes the cumulative percentage. **b** Observed velocity deficit as a function of upstream wind speed. Box plots show the inter-quartile range (25th-75th percentiles), with whiskers extending to the 20th and 80th percentiles. The red line indicates the mean deficit within each wind speed bin. *n *= 3789 samples.

The average near-surface velocity deficit of the remaining samples is 0.990 m/s, which corresponds to a 12.4% reduction compared to the upstream wind speeds. Figure 7a demonstrates that over half of the observed near-surface wind speed deficit are below 10%, while approximately 10% of the samples experience reductions exceeding 30%. Furthermore, Fig. 7b reveals a correlation between lower incoming wind speeds and increased wake-induced reductions, consistent with in-situ measurement results^55^ and may be related to the aerodynamic operating regime of wind turbines. Typically, wind turbines operate with a higher thrust coefficient at wind speeds below the rated value to maximize power extraction, which tends to generate more intense wakes. In contrast, at above-rated wind speeds, pitch control mechanisms reduce the thrust coefficient, potentially leading to a relative weakening of the wake intensity. The observed dependence of wake-induced wind speed deficit on upstream wind speed has important implications for long-term energy yield assessments. Because lower wind speed conditions are more susceptible to wake losses^56^, cumulative wake impacts may disproportionately affect annual energy production during moderate and low-wind regimes that contribute substantially to total operating hours. This nonlinear interaction suggests that average wake losses inferred from idealized or high-wind conditions may underestimate real-world, long-term impacts in densely populated wind farm regions.

**Fig. 8:**
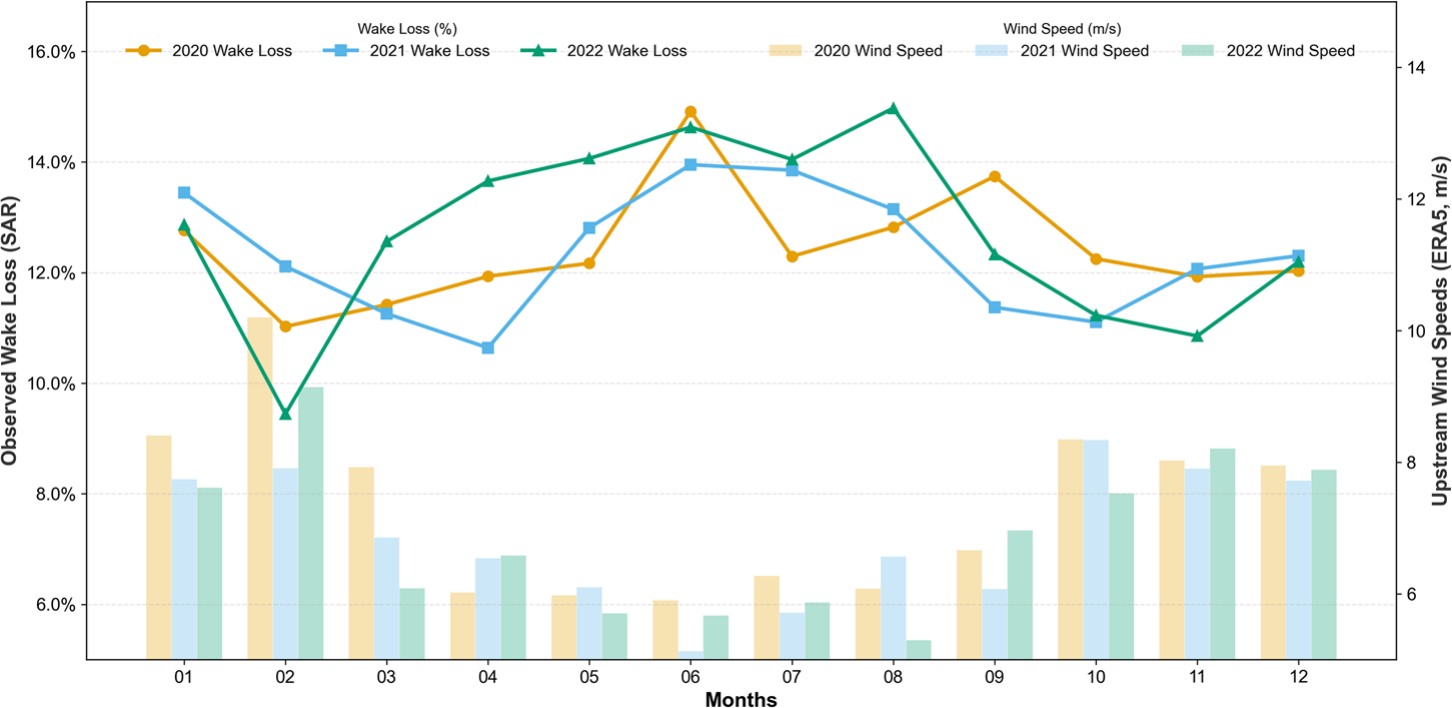
Monthly average wake-induced wind speed deficit. The horizontal axis represents the month. The line indicates the near-surface wind speed deficit observed by SAR, while the bars represent the corresponding monthly mean wind speed derived from ERA5.

Next, the monthly observed near-surface wake-induced wind speed deficits during the three years are presented in Fig. 8. The line graph shows that larger deficits are observed more frequently during the summer months compared to the winter months, which is consistent with previous studies^40^. To better interpret this seasonal pattern, we analyze the corresponding monthly mean background wind conditions using ERA5 hourly 10 m wind speed data^41^. It should be noted that ERA5 data are used only to characterize the seasonal background wind climate, while the wake-induced wind speed deficits themselves are derived entirely from SAR-retrieved 10 m wind speeds. The ERA5-derived monthly mean wind speeds over the study regions are shown as the bar graph in Fig. 8. The results indicate that the monthly mean wind speeds from April to September are generally lower than those from October to March. Considering the relationship between wind speed and wake deficit (Fig. 7b), where larger deficits tend to occur under lower ambient wind speeds, this seasonal variation in background wind conditions provides a plausible explanation for the more pronounced wake-induced deficits observed during the summer months.

### A first open-access SAR database for wind farm wakes

The SAR images used in this study have been made publicly available. The dataset is organized according to acquisition time and wind farm location. In addition, a curated collection of representative inter-farm wake interactions identified from SAR imagery is provided to facilitate further research on this phenomenon by both academia and industry. We believe that such a database can serve as a foundation for researchers to further develop and benchmark SAR-based wake detection and assessment frameworks, as well as to validate wind farm wake parameterizations used in mesoscale and engineering models. The dataset may also support the development of machine-learning approaches for automated wake detection and classification, and enable multi-sensor studies that combine SAR observations with other remote sensing or in-situ measurements. Furthermore, integrating this dataset with mesoscale simulations could help expand temporal coverage and improve wind speed extrapolation techniques from near-surface observations to turbine-relevant heights. Overall, the database is expected to benefit the research community investigating long-range wake signatures and improve our understanding of wake interactions among large offshore wind farm clusters.

At the same time, it should be noted that all wake metrics in this study are derived from SAR-retrieved 10 m neutral wind speeds and therefore represent near-surface wake signatures. Wind-farm-induced wake behavior may vary with height, and wake characteristics in the surface layer may differ from those in the turbine rotor layer (about 50–150 m), particularly under stable atmospheric conditions with strong vertical shear. Previous studies combining SAR observations and mesoscale modeling have shown that near-surface wind speed deficits or speed-up may coexist with classical wake deficits at hub height, reflecting stability-dependent turbulence redistribution and vertical momentum exchange^52^.

Consequently, long-range wake propagation observed at 10 m should not be interpreted as a direct proxy for turbine-level wake persistence or downstream power deficits. Instead, SAR-derived wake signatures provide valuable information on the large-scale, near-surface footprint of wind-farm-induced flow modification, which complements hub-height observations and modeling. Establishing quantitative relationships between near-surface and rotor-layer wake behavior remains an important topic for future research that requires coordinated satellite observations, in-situ measurements, and high-resolution numerical simulations.

### Uncertainty in SAR-derived wake deficit estimates

The CMOD5.N geophysical model function is known to exhibit different uncertainty characteristics depending on wind speed regime. At moderate wind speeds (approximately 4–15 m/s), CMOD5.N retrievals are generally robust, while increased uncertainty may occur under very low wind speeds due to reduced backscatter sensitivity and under high wind speeds due to signal saturation. In this study, extreme wind speed cases (<1 m/s and >25 m/s) were excluded from the quantitative analysis to reduce these effects. The remaining dataset predominantly lies within the well-validated operational range of CMOD5.N.

Table 2 summarizes a multi-year validation of Sentinel-1 SAR 10-m wind speeds retrieved using CMOD5.N against in-situ buoy observations. In addition to RMSE and mean bias, several complementary metrics are reported, including mean absolute error (MAE), correlation coefficient (*R*), and scatter index (SI), providing a more complete characterization of retrieval performance. To quantify statistical robustness, 95% confidence intervals for RMSE and bias were estimated using a bootstrap resampling approach. Across all buoy stations, RMSE values typically remain below approximately 1.0 m/s, with relatively narrow confidence intervals, indicating stable and consistent retrieval accuracy over different locations and sampling sizes. Correlation coefficients exceeding 0.9 further confirm the strong agreement between SAR-derived and in-situ wind speeds.

While Table 2 characterizes the absolute uncertainty of SAR-retrieved wind speeds, wake deficits in this study are defined as the difference between spatially averaged upstream and downstream wind speeds extracted from the same SAR acquisition. As a result, scene-level systematic errors in SAR wind retrieval are expected to partially cancel when computing the differential wake signal. The remaining uncertainty is therefore dominated by random retrieval noise, which is further reduced through spatial averaging over hundreds of pixels within the *R*^2^*G*^3^-identified regions. Consequently, the effective uncertainty of wake deficit estimates will be smaller than the absolute wind speed retrieval error reported in Table 2. And importantly, the reported mean wake deficit should be interpreted as a climatological average across thousands of independent SAR observations, rather than a deterministic estimate for individual wind farm pairs or specific meteorological conditions.

## Discussion

**Fig. 9:**
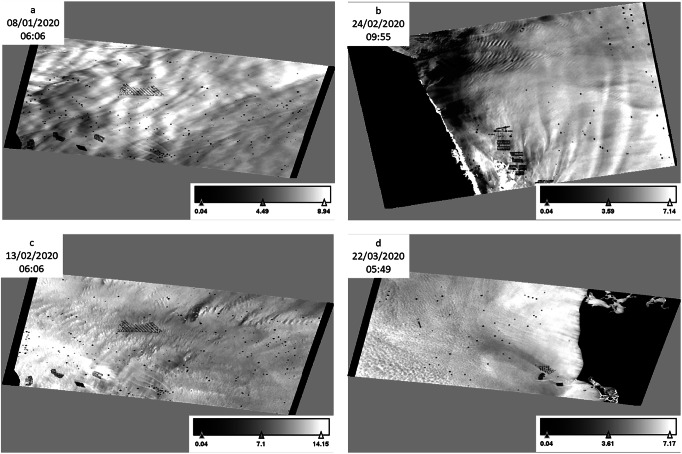
Examples of SAR-derived 10 m wind speed fields illustrating ambiguous boundaries between wake-affected and wake-free regions. The grayscale color bars represent SAR-retrieved neutral wind speed at 10 m height (m/s), with darker tones indicating lower wind speeds and lighter tones indicating higher wind speeds. The SAR scenes were captured: (**a**, **c**) near the coast of Holderness and Yorkshire; **b** in the Yellow Sea near Jiangsu Province; and **d** near the Danish Jutland coast. The acquisition date and time (UTC) are shown in the top-left corner of each subpanel. Credit of the source SAR images: European Space Agency (ESA).

Accurately identifying wakes in real-world SAR imagery, such as those shown in Fig. 9, remains a major challenge. Consequently, a thorough and precise assessment of long-range wake signatures demands further collective effort from the wider community. Looking ahead, several important directions are described below.

First, all current investigations are based on wind speeds at a height of 10 m, whereas typical turbine hub heights exceed 100 m. At present, there are no suitable tools or algorithms for extrapolating wind speed from 10 to 100 m. To overcome this challenge, it is important to collect in-situ wind speed measurements at 100 m in wake-affected regions and develop a practical algorithm to extend SAR-retrieved wind speeds to hub height.

Second, although the reduction in wind speed over distance caused by the wake is a key metric for wind farm planning, the irregular shape of the wake poses substantial measurement difficulties. Consequently, novel methods for measuring distance in this context will be an important area to enable SAR-supported wind farm planning.

Third, wind turbines operate under different performance regimes. For example, under above-rated wind conditions, power output is regulated and may remain approximately constant despite variations in wind speed, which can influence wake characteristics. Future research that incorporates turbine operational information could help better understand how different operating regimes affect wake intensity and downstream wind speed deficits.

Fourth, wake characteristics vary under different atmospheric stability conditions. While atmospheric stability indicators can be readily obtained from reanalysis products, systematically classifying and interpreting wake behavior under different stability regimes across thousands of SAR scenes remains methodologically challenging. Future studies combining stability metrics with automated wake characterization could provide a more comprehensive understanding of stability-dependent wake dynamics.

Finally, the proposed *R*^2^*G*^3^ metric still relies on manually selected parameters. The reason is that in real-world SAR imagery, even experienced experts often struggle to delineate the boundary between wake-free and wake-affected areas as it is not always clear and recognizable, such as the examples shown in Fig 9. In addition, turbine operational variability (e.g., temporary curtailment or partial loading) may reduce wake contrast in some cases, although such information is not directly observable from SAR data. A potential solution is to employ high-resolution simulations to model the wind field in the same region with and without turbine parameterization. Comparing these two scenarios could help more objectively delineate wake-affected areas and improve the robustness of wake identification.

To facilitate the use of the data from this paper, we will provide as much relevant information and data as possible for the wider communities, including wake labels generated by *R*^2^*G*^3^ as well as the parameters to generate the label, wake statistics for each SAR image, wind farm locations, turbine coordinates, SAR acquisition timestamps, and more. We envisage that these resources will lead to deeper insights into wake interactions among large-scale wind farms.

## Conclusions

This study confirms that near-surface wake signatures at 10 m height, as observed by SAR, can persist over distances exceeding 100 km in real-world offshore wind farm clusters. As a result, it suggests that the wake-free distance to guide new farm planning in the wind energy industry may need to be reexamined and revised. It should be emphasized that all results are based on SAR-retrieved 10 m neutral wind speeds. Although these near-surface wake signatures provide robust observational evidence of long-range wake influence, they cannot be directly extrapolated to turbine hub height or rotor-layer flow without additional modeling or in-situ measurements. Furthermore, this research represents the first international study to investigate 3789 cases using SAR-derived wind speed data. The study covers more than 60 wind farms across the globe and spans a three-year duration from 2020 to 2022. The results reveal that the average near-surface velocity deficit caused by wake effects is 0.990 m/s, equivalent to 12.4% of the upstream wind speeds. The substantial evidence obtained in this work provides strong observational support for the frequent and pronounced near-surface impact of wind farm wake signatures. This is particularly pronounced in sea areas densely populated with wind farm, such as the North Sea and Yellow Sea. These findings are expected to accelerate the progress of potential legislation and conflict resolution regarding wake-induced revenue losses between neighboring wind farms in the foreseeable future.

## Supplementary information

Supplementary Information

## Data Availability

The list of SAR images used in this study is available at https://github.com/lironui/SAR_WAKE.git. The SAR data are available at the Copernicus Open Access Hub, https://browser.dataspace.copernicus.eu/. The ERA5 data are available at the European Center for Medium-Range Weather Forecasts, https://www.ecmwf.int/en/forecasts/dataset/ecmwf-reanalysis-v5. The buoy data are available at the National Data Buoy Center, https://www.ndbc.noaa.gov. The SNAP toolbox is available at Science Toolbox Exploitation Platform, http://step.esa.int/main. The WRF model is available at https://github.com/wrf-model/WRF/releases.

## Code availability

The code for the proposed Restricted-Region Gradient-Guided Growing (*R*^2^*G*^3^) is available in the public repository at https://github.com/lironui/SAR_WAKE.git.
