# Supplementary material for: Long-range near-surface wake signatures of offshore wind farm clusters revealed by satellite observations
Source: Commun Eng. 2026 May 15;5:144. doi: 10.1038/s44172-026-00684-7 (PMC13429631; doi:10.1038/s44172-026-00684-7)
Supplement: Supplementary file 1 — Supplementary Information [file 44172_2026_684_MOESM1_ESM.pdf]

Supplementary Information for Long-range near-surface wake signatures of offshore wind farm clusters revealed by satellite observations

Rui Li, Jincheng Zhang, Xiaowei Zhao

Supplementary Note 1. R^2G^3 Algorithm Overview

The Restricted-Region Gradient-Guided Growing (R^2G^3) algorithm is designed to extract upstream reference regions and downstream wake-affected regions from SAR-retrieved wind speed fields in a consistent and scalable manner. The algorithm operates on a single SAR scene at a time and consists of four main steps: (i) wind farm localization and masking, (ii) upstream and downstream search region definition, (iii) gradient-constrained region growing for wake identification, and (iv) quality control and filtering.

Table 5 summarizes the default parameter values used in the R^2G^3 algorithm. Unless otherwise stated, these parameters are applied uniformly to all SAR scenes analyzed in this study to ensure methodological consistency between wind farms, regions, and seasons. The wind farm radius R is defined as the maximum distance from the farm centroid to the outermost turbine and is measured once for each wind farm based on turbine layout. This parameter is independent of SAR imagery and meteorological conditions and therefore does not introduce scene-specific tuning. Certain parameters in the R^2G^3 algorithm are defined adaptively to account for scene-to-scene variability in SAR-derived wind fields. For example, the gradient threshold G_T is computed as a multiple of the mean wind speed gradient estimated from pixels within a predefined gradient range (0.1–50), excluding turbine-contaminated areas. This adaptive definition allows the algorithm to adjust to variations in overall wind speed level and SAR noise while maintaining a consistent relative sensitivity across all scenes.

Importantly, parameter values were selected based on empirical inspection of representative SAR scenes and were fixed prior to large-scale analysis to avoid scene-specific tuning.

Supplementary Note 2. R^2G^3 Parameter Sensitivity

To assess the robustness of the R^2G^3 algorithm to user-defined parameter choices, a sensitivity analysis was conducted by systematically varying key parameters, including the upstream length, downstream length, and the lower and upper wake speed ratio thresholds, while keeping all other parameters fixed at their default values. For each configuration, wake deficits were recomputed across the full multi-year dataset, and the resulting mean wake speed ratio and corresponding 95% confidence intervals were recorded.

For the downstream length, values ranging from 30 to 100 km were examined. The mean wake speed ratio shows a gradual and monotonic increase with increasing downstream length; however, variations remain small and the 95% confidence intervals largely overlap across the tested range. This indicates that the algorithm is not highly sensitive to the precise choice of downstream length, provided it is sufficiently large to capture the undisturbed inflow region.

Similarly, the upstream length was varied between 5 and 15 km. The resulting mean wake speed ratios and confidence intervals show only minor differences, suggesting that the wake extraction is robust to reasonable changes in the upstream extent used for wake characterization.

The sensitivity to the lower wake speed ratio threshold was tested for values between 0.5 and 0.7. The results indicate stable performance for thresholds of 0.5 and 0.6, while a slightly lower mean value is obtained for 0.7, reflecting the exclusion of weaker wake signals. Nevertheless, the overall variations remain limited, demonstrating that the algorithm performance is not dominated by the exact choice of this threshold.

For the upper wake speed ratio threshold, a wider sensitivity is observed. A low upper threshold (1.05) leads to a pronounced increase in the mean wake deficit, as the stricter criterion tends to retain only pixels with stronger wind speed reductions. In contrast, thresholds of 1.10 and 1.15 yield consistent results, with overlapping confidence intervals. This supports the use of an upper threshold of 1.10 as a balanced choice between wake detection sensitivity and noise rejection.

Overall, the parameter sensitivity analysis demonstrates that the R^2G^3 algorithm is robust to reasonable variations in its key parameters. The default parameter set adopted in this study lies within statistically stable regions of the parameter space, ensuring that the reported wake characteristics are not artifacts of specific parameter choices.

Supplementary Note 3. Physical Consistency Assessment

To assess the physical plausibility of the long-range and transboundary wake signatures identified in SAR imagery, mesoscale simulations were performed using the Weather Research and Forecasting (WRF) model (v4.6.1)[1]. The objective is to evaluate whether a physical numerical framework reproduces the observed cross-boundary wake propagation under similar meteorological conditions.

WRF was configured with three nested domains at 27, 9, and 3 km horizontal resolution. The innermost 3 km domain covers the offshore wind farm clusters. We employed 62 vertical levels with enhanced resolution in the lower troposphere (the first level is about 10 m above ground level) to accurately resolve boundary-layer interactions and turbine-induced momentum extraction. Boundary layer processes were parameterized using the Mellor-Yamada-Nakanishi-Niino (MYNN) Level 2.5 scheme, which is well-suited for simulating turbine-induced

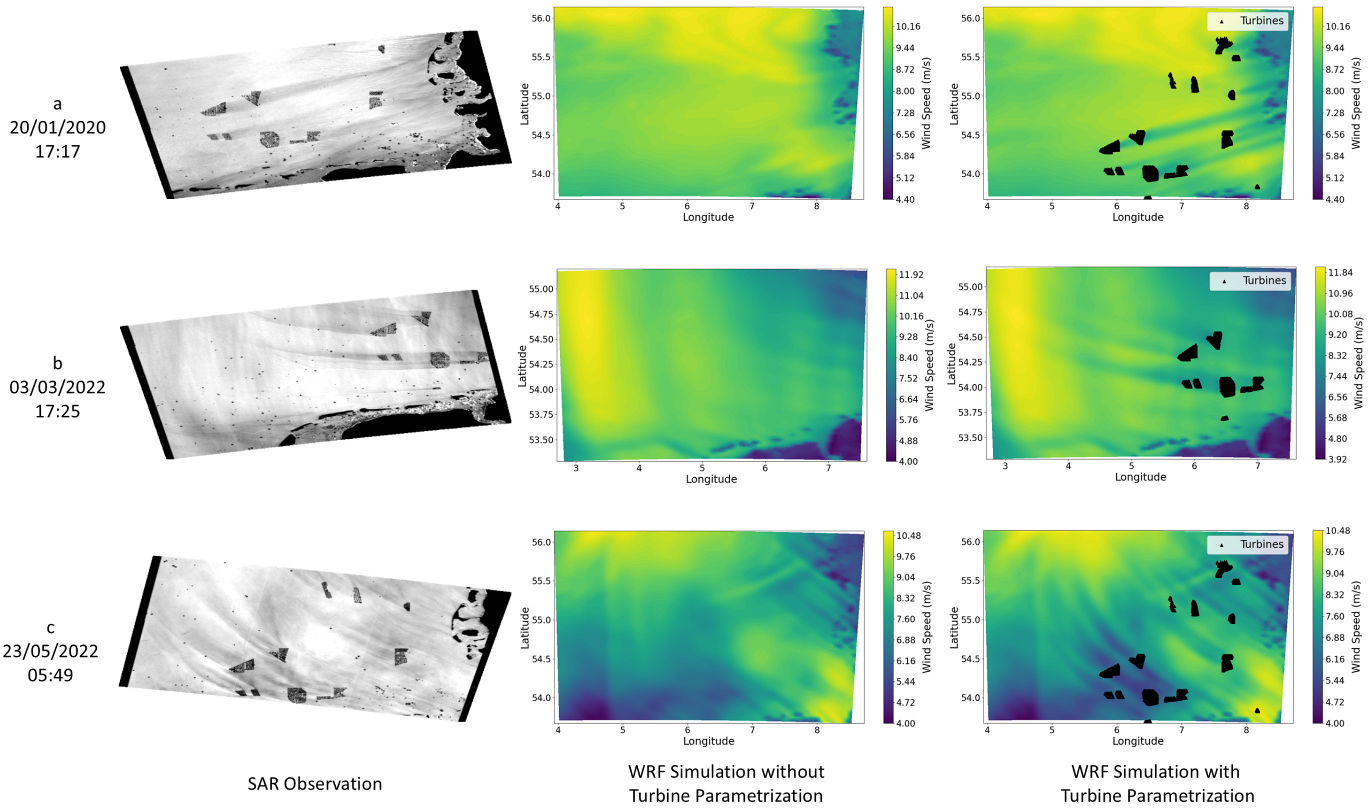

Supplementary Figure 1: **Validation of simulated long-range and transboundary wakes using SAR observations and WRF sensitivity simulations.** Rows (a–c) show three representative cases on 20 January 2020 (17:17), 3 March 2022 (17:25), and 23 May 2022 (05:49), respectively. The first column presents Sentinel-1 SAR observations. The second column shows WRF simulations without wind farm parameterization, and the third column shows WRF simulations with wind farm parameterization. Color shading represents wind speed, and black regions indicate wind turbine locations. The inclusion of wind farm parameterization in WRF enables the simulation of wake effects, which are compared with SAR-observed wake structures. Credit of the source SAR images: European Space Agency (ESA).

turbulence. Other standard physics include the Thompson microphysics, the Rapid Radiative Transfer Model for General Circulation Models (RRTMG) radiation scheme, and the Noah Land Surface Model (LSM). Initial and boundary conditions were derived from ERA5 reanalysis data[2] at 3-hour intervals, with spectral nudging applied in the outer domain to maintain large-scale atmospheric fidelity. We implemented the Ma et al. (2022) scheme[3], which incorporates the XA (Xie-Archer) wake model[4] and a sophisticated hub-height wind speed superposition method. This approach accounts for subgrid-scale wake interactions within farm clusters, providing an alternative representation of subgrid-scale wake interactions compared with conventional linear superposition approaches. Turbine specifications and locations were identical to those used in the SAR analysis. Two simulations were performed for each selected case: a control simulation without turbine parameterization and a wind farm simulation with turbine parameterization activated. All other model settings were kept identical between the two experiments, enabling direct comparison of wind fields with and without wind farm effects.

As demonstrated by the contrast between the control and turbine-parameterized simulations in Fig. 1, elongated low-wind-speed structures emerge only when wind farm parameterization is activated. In the control simulations, no coherent downstream wake structures are evident at comparable scales. The spatial alignment between SAR-retrieved wake signatures and WRF-simulated wake structures provides independent dynamical support for the physical plausibility of the observed long-range and transboundary wake propagation. While differences in magnitude and fine-scale structure are expected due to model resolution (3 km), parameterization assumptions, and the inherent differences between grid-averaged model output and SAR-retrieved 10 m neutral winds, the consistency in propagation direction and spatial extent indicates that the satellite-observed wake features are unlikely to arise solely from satellite sampling geometry. These results suggest that as offshore wind farm clusters expand in scale and density, their wake influence may extend across jurisdictional boundaries under favorable atmospheric conditions. This highlights the potential relevance of coordinated maritime spatial planning and cross-border resource management, particularly in semi-enclosed basins such as the North Sea.

While the present simulations demonstrate physical consistency for selected case studies, a comprehensive climatological assessment of transboundary wake frequency would require long-term multi-year simulations coupled with directional statistics, which we identify as an important direction for future work.

Supplementary Table 1: Sensitivity of the R^2G^3 algorithm to the downstream length parameter.

Downstream length	Mean	95% CI (L)	95% CI (H)
30	0.902	0.874	0.930
40	0.958	0.930	0.986
50	0.990	0.961	1.018
60	1.008	0.980	1.037
70	1.020	0.991	1.049
80	1.033	1.004	1.063
90	1.041	1.011	1.070
100	1.043	1.014	1.073

Supplementary Table 2: Sensitivity of the R^2G^3 algorithm to the upstream length parameter.

Upstream length	Mean	95% CI (L)	95% CI (H)
5	0.979	0.948	1.010
10	0.990	0.961	1.018
15	1.012	0.983	1.041

References

[1] William C Skamarock, Joseph B Klemp, Jimy Dudhia, David O Gill, Zhiquan Liu, Judith Berner, Wei Wang, Jordan G Powers, Michael G Duda, Dale M Barker, et al. A description of the advanced research wrf version 4. *NCAR tech. note ncar/tn-556+ str*, 145, 2019.

[2] Hans Hersbach, Bill Bell, Paul Berrisford, Gionata Biavati, András Horányi, Joaquín Muñoz Sabater, Julien Nicolas, Carole Peubey, Raluca Radu, Iryna Rozum, et al. Era5 hourly data on single levels from 1979 to present. *Copernicus climate change service (c3s) climate data store (cds)*, 10(10.24381), 2018.

[3] Yulong Ma, Cristina L Archer, and Ahmadreza Vasel-Be-Hagh. The jensen wind farm parameterization. *Wind Energy Science*, 7(6):2407–2431, 2022.

[4] Shengbai Xie and Cristina Archer. Self-similarity and turbulence characteristics of wind turbine wakes via large-eddy simulation. *Wind Energy*, 18(10):1815–1838, 2015.

Supplementary Table 3: Sensitivity of the R^2G^3 algorithm to the lower wake speed ratio threshold.

Lower wake speed ratio	Mean	95% CI (L)	95% CI (H)
0.5	0.990	0.962	1.018
0.6	0.990	0.961	1.018
0.7	0.956	0.928	0.983

Supplementary Table 4: Sensitivity of the R^2G^3 algorithm to the upper wake speed ratio threshold.

Upper wake speed ratio	Mean	95% CI (L)	95% CI (H)
1.05	1.142	1.102	1.182
1.10	0.990	0.961	1.018
1.15	0.949	0.922	0.977
